# Characterization of a secreted cystatin of the parasitic nematode *Haemonchus contortus* and its immune-modulatory effect on goat monocytes

**DOI:** 10.1186/s13071-017-2368-1

**Published:** 2017-09-18

**Authors:** Yujian Wang, Lingyan Wu, Xinchao Liu, Shuai Wang, Muhammad Ehsan, RuoFeng Yan, XiaoKai Song, LiXin Xu, XiangRui Li

**Affiliations:** College of Veterinary Medicine, Nanjing Agricultural University, Nanjing, People’s Republic of China

**Keywords:** *Haemonchus contortus*, Cystatin, Monocyte, Immunomodulation

## Abstract

**Background:**

Haemonchosis is a disease of the small ruminant caused by a nematode parasite *Haemonchus contortus*, and it is most important and alarming challenges to the small ruminant’s production. The infection of the *H. contortus* could cause high economic losses worldwide. *H. contortus* is a blood feeding parasite which penetrates into the abomasal mucosa to feed the blood of the host and causing the anemia and decreased total plasma protein. Modulation and suppression of the immune response of the host by nematode parasites have been reported extensively, and the cysteine protease inhibitor (cystatin) is identified as one of the major immunomodulators.

**Methods:**

The recombinant protein of HCcyst-3 was expressed in a histidine-tagged fusion soluble form in *Escherichia coli*, and its inhibitory activity against cathepsin L, B, as well as papain, were identified by fluorogenic substrate analysis. Native HCcyst-3 protein was localized by an Immunohistochemical test. The immunomodulatory effects of HCcyst-3 on cytokine secretion, MHC molecule expression, NO production and phagocytosis were observed by co-incubation of rHCcyst-3 with goat monocytes.

**Results:**

We cloned and produced recombinant cystatin protein from *H. contortus* (rHCcyst-3) and investigated its immunomodulatory effects on goat monocyte. The rHCcyst-3 protein is biologically functional as shown by its ability to inhibit the protease activity of cathepsin L, cathepsin B, and papain. The immunohistochemical test demonstrated that the native HCcyst-3 protein was predominantly localized at the body surface and internal surface of the worm’s gut. We demonstrated that rHCcyst-3 could be distinguished by antisera from goat experimentally infected with *H. contortus* and could uptake by goat monocytes. The results showed that the engagement of rHCcyst-3 decreased the production of TNF-α, IL-1β and IL-12p40. However, it significantly increased the secretion of IL-10 and TGF-β1 in goat monocytes. After rHCcyst-3 exposure, the expression of MHC-II on goat monocytes was restricted. Moreover, rHCcyst-3 could upregulate LPS induced NO production of goat monocytes. Phagocytotic assay by FITC-dextran internalization showed that rHCcyst-3 inhibited the phagocytosis of goat monocytes.

**Conclusions:**

Our results suggested that the recombinant cystatin from *H. contortus* (rHCcyst-3) significantly modulated goat monocyte function in multiple aspects.

**Electronic supplementary material:**

The online version of this article (10.1186/s13071-017-2368-1) contains supplementary material, which is available to authorized users.

## Background

*Haemonchus contortus* is one of the most economically important parasites of small ruminants worldwide. Infection can lead to anaemia, loss of condition and death of the host, especially lambs [1, 2]. The cystatin superfamily consists of evolutionary related reversibly, tight-binding inhibitors of papain-like cysteine proteases [3]. Cystatins are classified, based on characteristic sequence motifs and the number of conserved cystatin domains, into four subfamilies: the type 1 cystatins (also known as stefins), type 2 cystatins, type 3 cystatins (kininogens), and the type 4 cystatin-like proteins (fetuins and histidine-rich proteins) [4]. Type 1 cystatins are cytoplasmic proteins that do not have signal peptides; however, the type 2 cystatins are secretion-type proteins containing signal peptides. Cystatins are present in a wide range of organisms, such as vertebrates, invertebrates, plants and as well as protozoa [5, 6]. They are involved in various vertebrate biological processes, such as antigen presentation, immune system development, epidermal homeostasis, neutrophil chemotaxis during inflammation and apoptosis [7–10].

Parasitic nematodes, living in the intestinal tract or within tissues of their hosts, are constantly exposed to an array of immune effector mechanisms. One strategy to cope with the immune response is the release of immunomodulatory components that block effector mechanisms or interact with the cytokine network [11]. The sophistication of mammalian innate and adaptive immune systems and the long co-evolutionary relationship between host and parasite, both imply that a considerable number of molecular interactions are in play [12]. Some studies in recent years have shown that cystatins are one of the major immune modulators produced by nematode parasites [6, 13]. Cytokine secretion, MHC molecule expression, NO production and phagocytosis were very important to monocytes to exercise its immune function.

Here, we cloned a cystatin gene from *H. contortus*, produced the recombinant protein and analyzed for its immune modulatory activity. We observed that the recombinant cystatin from *H. contortus* (rHCcyst-3) significantly modulated goat monocyte function in multiple aspects.

## Methods

### Parasites and animals

The *H. contortus* strain (designated Nanjing 2005) was originally obtained from Nanjing (Jiangsu Province, China) and maintained by serial passage in 3–6-month-old, helminth-free goats [14]. Third stage larvae (L3) used for the challenge were cultured from the feces of the monospecifically infected goats at 26 °C and stored in water at a concentration of 2500 larvae/ml at 4 °C.

Local crossbred male goats (3–6-month-old) from the teaching and research flock at Nanjing Agricultural University were housed indoors in pens containing six goats per pen. The goats were fed hay and whole shelled corn and provided with water ad libitum. All goats were dewormed twice at 2-week intervals with levamisole (8 mg/kg body weight) orally at the time of housing to remove naturally acquired strongylid infection. After 2 weeks, a fecal sample from each goat was examined by microscope for helminth eggs, according to standard parasitological techniques. Goats exhibiting no eggs were used in the subsequent study, and daily health observations were performed throughout the experiment.

SD rats (body weight ~ 150 g) were purchased from Experimental Animal Center of Jiangsu, PR China (Qualified Certificate: SCXK 2008–0004) and were raised in a sterilized room and fed sterilized food and water.

### Cloning of HCcyst-3 and bioinformatics analyses

Utilizing resources from online database, the open reading frame (ORF) of cystatin-like gene (GenBank: CDJ92568.1) without signal peptide sequence was amplified by reverse transcription-polymerase chain reaction (RT-PCR) using designed specific primers (forward primer: 5′-TAG AAT TCG GTA TGG TCG GAG GAT TTA-3′ and reverse primer: 5′-TAC TCG AGG ACC TGC TCT CCT TCA GCG-3′), in which the *EcoR*I and *Xho*I restriction sites, respectively, were introduced and are shown underlined. Following ligation of the obtained RT-PCR product with the pMD19-T vector (Takara, Dalian, China) to form pMDcystatin, the cystatin fragment was cleaved from pMDcystatin with *EcoR*I and *Xho*I and subcloned into the corresponding sites of pET32a vector (Invitrogen, Carlsbad, CA, USA). The accuracy of the insertion in the resulting plasmid was confirmed by sequencing.

### Expression and purification of rHCcyst-3 in *Escherichia coli*

The expression of the recombinant fusion protein in *E. coli* BL-21 cells (DE3) was induced by isopropyl-β-D-thiogalactopyranoside (IPTG) at a final concentration of 1 mM for 6 h at 37 °C in Luria-Bertini (LB) medium with ampicillin (100 μg/ml). The histidine-tagged fusion protein was purified from the supernatant of bacterial lysates using the His•Bind® 128 Resin Chromatography kit (Novagen, Madison, USA), according to the manufacturer’s instructions, and dialyzed in phosphate buffered saline (PBS, pH 7.4) to remove imidazole. The empty pET32a was used for producing control histidine-tagged protein, which was expressed and purified identically to the procedure for the cystatin-histidine-tagged fusion protein. The purity of the purified rHCcyst-3 was analyzed by 12% sodium dodecyl sulfate polyacrylamide gel electrophoresis (SDS-PAGE) followed by Coomassie blue staining. Protein concentrations were determined by Bradford method. LPS was depleted from the rHCcyst-3 using Detoxi-Gel Affinity Pak prepacked columns (Thermo Fisher Scientific, Waltham, MA, USA). The concentrations of the recombinant proteins were equalized to 1 mg/ml before LAL assay. Endotoxin levels of the protein samples were measured by LAL gel clot assay using a Pyrosate® Kit (Cape Cod Inc., East Falmouth, MA, USA). The samples whose endotoxin content was less than the sensitivity of the Pyrosate kit (< 1 EU per 1 mg of the recombinant proteins) were collected for the subsequent experiments.

### Generation of polyclonal antibodies

The goat antisera used in western blot analyses were collected from five goats experimentally infected with *H. contortus*. The goats were raised in helminth-free conditions and then orally challenged with 5000 infective L3. One month later, the goat antisera were collected and stored at -70 °C until use.

To generate polyclonal antibodies against rHCcyst-3, 0.3 mg of purified rHCcyst-3 was mixed with Freund’s complete adjuvant (1:1) and injected into SD rats subcutaneously in multiple places, following the method described by Han et al. [15]. After the first injection, rats were then boosted four times at 2-week intervals with the same dose. The sera containing specific anti-rHCcyst-3 antibodies were harvested 10 days following the last injection, and the specific reactivity with rHCcyst-3 was checked by enzyme-linked immunosorbent assay (ELISA).

### Western blot analysis

Purified rHCcyst-3 (20 μg) was resolved on 12% SDS-PAGE and transferred to Hybond-C extra nitrocellulose membranes (Amersham Biosciences, London, UK). Non-specific binding sites were blocked by immersing the membranes in 5% skim milk in Tris-buffered saline (TBS) for 1 h at room temperature. The membranes were then washed 5 times (5 min each) in TBS containing 0.1% Tween-20 (TBST). Subsequently, the membranes were incubated with the primary antibodies (antiserum from goats experimentally infected with *H. contortus*) for 1 h at 37 °C (dilutions 1:100 in TBST). After being washed 5 times with TBST, the membranes were then incubated with HRP-conjugated rabbit anti-goat IgG (Sigma, St. Louis, MO, USA) for 1 h at 37 °C (diluted 1:2000 in TBST). Finally, the immunoreaction was visualized using freshly prepared diaminobenzidine (DAB, Sigma) as a chromogenic substrate after 5 min.

### Localization of HCcyst-3 by immunohistochemical study

Washed adult worms suspended in PBS were fixed in 4% formaldehyde-0.2% glutaraldehyde in PBS for 90 min and then immersed in TISSUE-TeK® O.C.T. compound (SAKURA, Torrance, CA, USA). They were snap frozen in liquid nitrogen and stored at −20 °C until required for further processing. Cryostat sections of 10 μm thickness were cut, washed with PBS, and treated for 60 min with 10% normal goat serum in PBS to prevent non-specific binding of antibodies. The sections were then incubated with specific rat anti-rHCcyst-3 antiserum (1:100 dilution) or normal rat serum (control) for 60 min at 37 °C, washed 15 min × 3 with PBS, and subsequently incubated for 60 min with Cy3 goat anti-rat IgG (ab6953, Abcam, Cambridge, MA, USA). Finally, the sections were stained with DAPI (Beyotime, Haimen, Jiangsu, China) to show DNA. After washing with PBS, the specimens were immersed in Anti-Fade Fluoromount solution (Beyotime), which prevents fading of fluorescence during microscopic examination.

### Proteinase inhibition assays

To calculate the inhibitory activity of the recombinant protein, the concentration of rHCcyst-3 at which a 50% inhibition of the proteolytic enzymes’ activities was achieved (IC50) and measured [16]. Recombinant protein was preincubated with each enzyme in an assay buffer for 30 min. Then, 0.25 mM of the protease-specific substrates was added to each well, and residual enzyme activity monitored. The histidine-tagged protein was used as a control. Enzymes used were as follows: human cathepsin L (0.05 μM), human cathepsin B (0.05 μM), human caspase 1 (0.05 μM), as well as papain (0.15 μM). All of these enzymes were purchased from Sigma Company. The assay buffer used consisted of 100 mM sodium acetate, pH 5.5, 100 mM NaCl, 1 mM EDTA, 1 mg/ml cysteine, and 0.005% TritonX-100. The substrates purchased (Sigma-Aldrich) were as follows: Z-Phe-Arg-AMC·HCl for papain, cathepsin L and cathepsin B; and Ac-Tyr-Val-Ala-Asp-AFC for caspase 1. Fluorescence intensity was monitored by SPECTRAFLUOR (TECAN, Maennedorf, Switzerland) with the wavelength pair of 360–460 nm for emission and excitation, respectively.

### Isolation of goat monocytes

Peripheral blood mononuclear cells (PBMCs) were separated from heparinized blood with the standard Ficoll-hypaque (GE Healthcare, Little Chalfont, USA) gradient centrifugation method and washed twice in PBS. Monocytes were isolated by their adherence to the plastic surface [17]. The goat PBMCs were seeded in a 6 wells flat-bottom tissue culture plates (Corning, New York, USA) in cell culture medium RPMI 1640 (GIBCO, New York, USA) containing 10% heat inactivated fetal calf serum (GIBCO), 100 U/ml penicillin and 100 mg/ml streptomycin (GIBCO). Plates were incubated at 37 °C in a humidified atmosphere with 5% CO_2_ for 1 h [18]. Non-adherent cells were removed by washing twice with PBS. The adherent cells were collected and adjusted to a density of 1 × 10^6^ cells/ml in cell medium at 37 °C in a humidified atmosphere with 5% CO_2_. Cell viability, as determined by trypan blue dye exclusion, was more than 95% in all cases.

### Uptake the rHCcyst-3 by goat monocytes

Freshly isolated goat monocytes were seeded into 24-well plates with rHCcyst-3 (40 μg/ml). The non-treated cells were set as control. After incubation at 37 °C for 30 min, cells were collected and washed twice with ice-cold PBS. The IF analyses were performed on 4% paraformaldehyde-fixed monocytes (rHCcyst-3 treatment and control) plated on 0.01% poly-L-lysine-coated cover slips, then, cells were permeabilized by incubation for 5 min in 0.5% Triton X-100 in PBS, and were treated with a blocking solution (2% BSA in PBS) for 30 min to reduce background staining. After sequential incubation with specific rat anti-rHCcyst-3 antiserum (1:100 dilutions), for 2 h and incubation with Cy3 goat anti-rat IgG (ab6953, Abcam) for 1 h, DIO and DAPI (Beyotime) were used for plasma membrane and nucleus staining, respectively, for 6 min each. Then, protein localization was determined by observing the staining patterns with a 100 × oil objective lens on a laser scanning confocal microscope (LSM710, Zeiss, Jena, Germany). Exposure conditions were applied uniformly for each color channel. All procedures were carried out at room temperature. Digital images were captured using the Zeiss microscope software package ZEN 2012 (Zeiss).

### Detection of cytokine secretion

To determine cytokine secretion, goat monocytes were stimulated with LPS (100 ng/ml) for 72 h in the presence or absence of rHCcyst-3. The supernatants were collected, and cytokine testing was performed by ELISA. The levels of TNF-α, IL-1β, IL-10, IL-12p40 and TGF-β1 in supernatants were determined using commercially available goat ELISA kits (Anoric, Tianjin, China). The analysis was performed with the data from three independent experiments.

### Analysis of MHC molecule expression

The purified monocytes (0.5 × 10^6^ cells/ml) were incubated with different concentrations of rHCcyst-3 or equal volumes of control buffer for 24 h in complete RPMI 1640 at 37 °C. Cells were then stained with the monoclonal antibodies to MHC-I (MCA2189A647, AbD serotec, BioRad Laboratories, CA, USA) and MHC-II (MCA2226F, AbD serotec), and analyzed on a FACS Calibur cytometer (BD Biosciences, San Jose, CA, USA). Results were expressed as the percentage of mean fluorescence intensity (MFI) of control.

### Measurement of nitric oxide production

To determine nitric oxide production, goat monocytes were stimulated with LPS (100 ng/ml) for 48 h in the presence or absence of rHCcyst-3.NO was measured in the cell supernatants as nitrite using a NO assay kit (Beyotime), according to the manufacturer’s protocol. Briefly, a standard curve was prepared with standard nitrite solutions in DMEM medium. The standard solutions or cell supernatants were reacted with nitrate reductase for 30 min in a 96-well plate, and then Griess reagent I and Griess reagent II were added. After incubation at room temperature for 10 min, the absorbance at 540 nm was read in a microplate reader (BioRad). The samples were assayed in triplicate.

### FITC-dextran internalization

To confirm the effect of rHCcyst-3 on the phagocytotic ability of goat monocytes, the FITC-dextran internalization of cells was analyzed by flow cytometry [19]. Cells were collected after treated with rHCcyst-3 for 48 h and incubated with FITC-dextran (1 mg/ml in RPMI1640) for 1 h at 37 °C. Cells added with the same amount of FITC-Dextran and incubated at 4 °C for 1 h were used as the baseline of monocyte phagocytosis. After incubation, cells were washed extensively to remove excess FITC-dextran. The FITC-dextran internalization of cells was analyzed by flow cytometry (BD Biosciences) using Cell Quest Software, and median fluorescence intensity (MFI) was calculated.

### Statistical analysis

Data are expressed as mean ± the standard deviation of the mean. Statistical analysis for significant differences was performed using an analysis of variance, the Student’s *t*-test for parametric samples (GraphPad Prism, USA).

## Results

### Cloning and sequence analysis of HCcyst-3

**Fig. 1:**
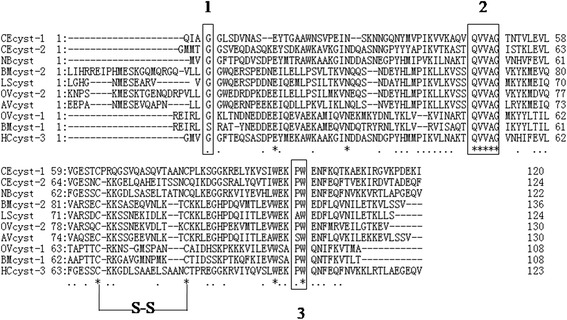
Putative amino acid alignment of HCcyst-3 with another nematode type 2 cystatins: CEcyst-1 of *Caenorhabditis elegans*(GenBank: AF100663); CEcyst-2 of *Caenorhabditis elegans*(GenBank: AF068718); NBcyst of *Nippostrongylus brasiliensis*(GenBank: AB050883); BMcyst-1 of *Brugia malayi* (GenBank: U80972); BMcyst-2 of *Brugia malayi* (GenBank: AF015263); LScyt of *Litomosoides sigmodontis* (GenBank: AF229173); OVcyst-1 of *Onchocerca volvulus* (GenBank: AF177194); OVcyst-2 of *Onchocerca volvulus*(GenBank: P22085); and AVcyst of *Acanthocheilonema viteae* (GenBank: L43053). The predicted signal peptide of each sequence was excluded. The conserved cystatin active sites are boxed (1: N-terminal conserved glycine; 2: QXVXG conserved motif; 3: PW conservative site)

While searching the online database, a cystatin homologue was identified and cloned from *H. contortus*, designated HCcyst-3. It contains 143 amino acid residues with a predicted molecular mass of 13.5 kDa and an isoelectric point of 5.62. A sequence analysis demonstrated that HCcyst-3 possesses a conserved N-terminal glycine, QXVXG and PW motifs, and a disulfide bridge, which is highly conserved in various type 2 cystatins (Fig. 1). SMART analysis (http://smart.embl-heidelberg.de/) detected the cystatin-like domain in the putative amino acid sequence (position 21–137). A BLASTP analysis of the predicted polypeptide sequence against all non-redundant databases accessed through GenBank revealed significant similarity scores with members of the cystatin type 2 family of other nematodes. The amino acid analysis using the Signal P program revealed the presence of an obvious signal peptide which the cleavage site was predicted between amino acids 20 and 21.

### Expression and purification of rHCcyst-3

**Fig. 2:**
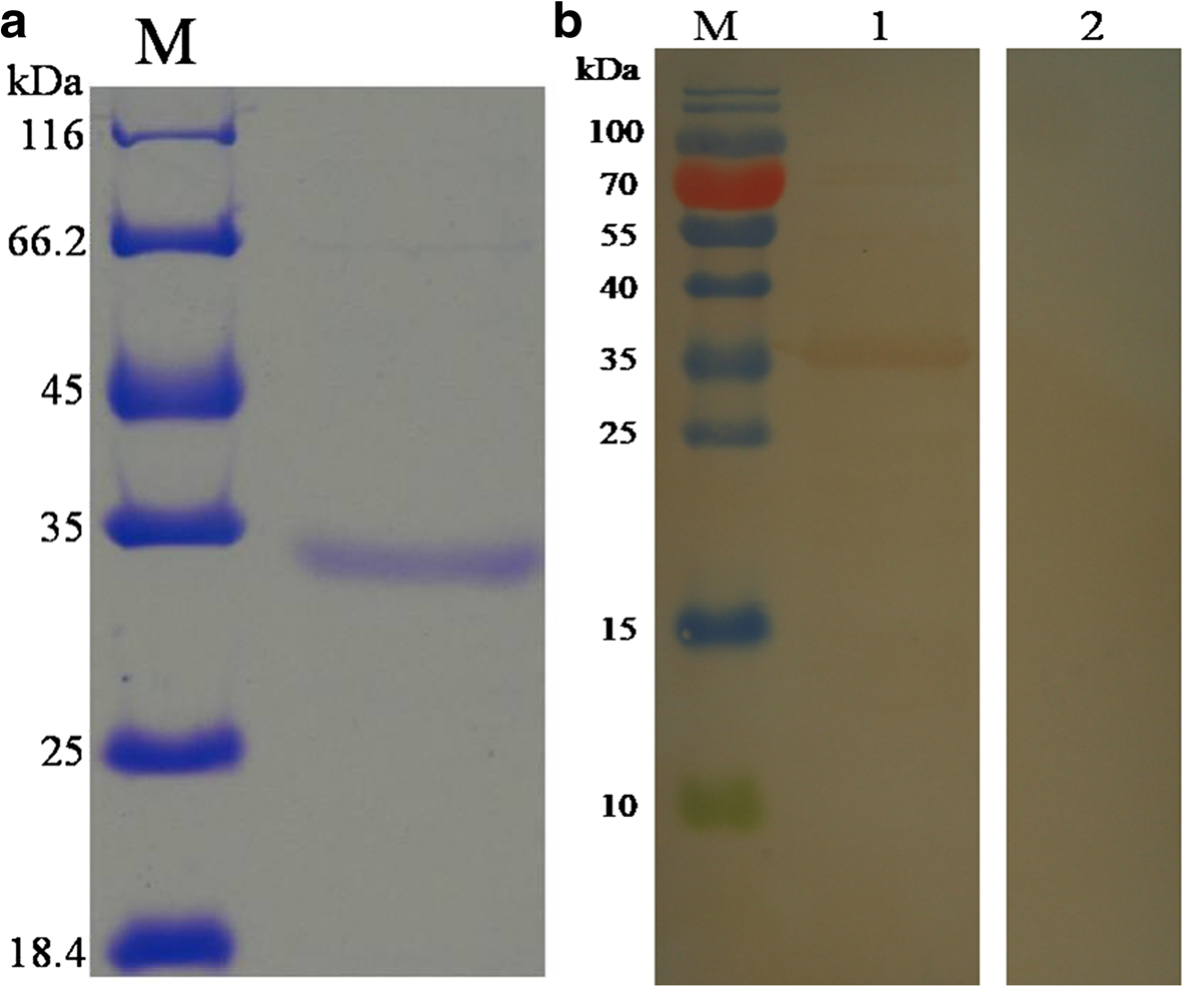
Purification of rHCcyst-3 and western blot. **a** Purified rHCcyst-3 were resolved by SDS-PAGE on 12% of polyacrylamide gel and stained with Coomassie brilliant blue R250. **b** Western blot analysis of Purified rHCcyst-3. Proteins are recognized by sera from goats experimentally infected with *H. contortus* as primary antibody (Lane 1) and normal goat (Lane 2) as the control

The gene encoding HCcyst-3 was ligated into the bacterial expression vector pET32a, and the recombinant protein was successfully expressed as a double His 6 tagged fusion protein with an expected size of 35 kDa (Fig. 2a). The rHCcyst-3 was expressed in a soluble form and then purified by affinity chromatography using the His•Bind® 128 Resin Chromatography kit (Novagen), according to the manufacturer’s instructions. The purity of rHCcyst-3 was more than 95% as estimated by SDS-PAGE analysis.

### Western blot analysis

To determine whether the HCcyst-3 protein was exposure to host immune system, serum from goats experimentally infected with *H. contortus* was used as a primary antibody to react with rHCcyst-3. Western blot analysis showed that the antiserum could recognize rHCcyst-3 (Fig. 2b), suggesting that HCcyst-3 could exposure to host immune system during the parasitic process.

### Immunolocalization of HCcyst-3

**Fig. 3:**
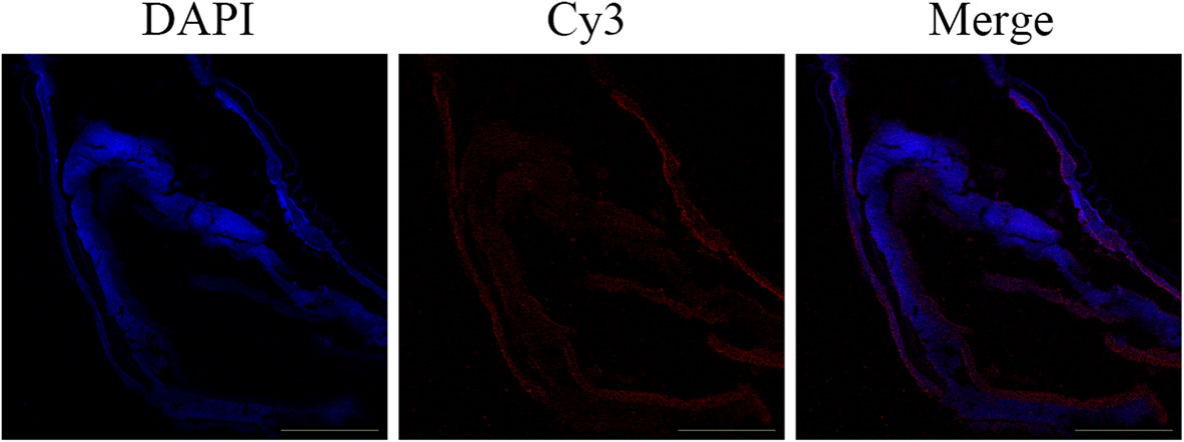
Immunohistochemical localization of the HCcyst-3 protein in a cryostal section of *H. contortus.* HCcyst-3 protein was detected by the indirect immunofluorescence method using second antibody Cy3 labeled goat anti-rat IgG (ab6953, Abcam). The section was counterstained with DAPI to show DNA. *Scale-bars*: 100 μm

A section through a partial body length of an adult female worm was shown in Fig. 3. HCcyst-3 and DNA fluorescence red and blue, respectively. The antibody eluted from rHCcyst-3 bound predominantly to the body surface, and the internal surface of the parasite’s gut (Fig. 3) and no labeling was observed in control experiments. The results show that HCcyst-3 is an excretory/secretory antigen.

### Proteinase inhibition assays

**Fig. 4:**
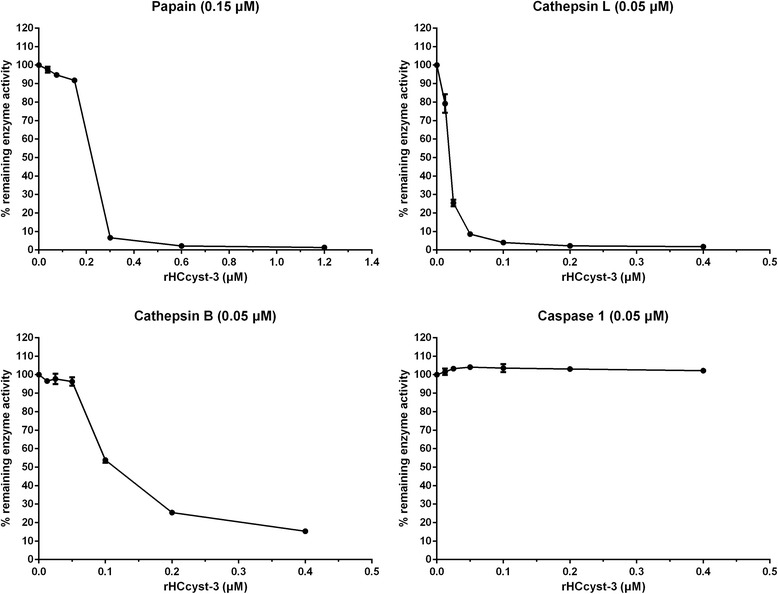
Inhibition of protease activities by the recombinant protein of HCcyst-3. Cathepsin L, cathepsin B, papain and caspase 1 were incubated with each of the substrates in the presence of different concentrations of rHCcyst-3. Incubation of proteases without rHCcyst-3 resulted in 100% enzyme activity

To investigate the efficiency of HCcyst-3 in inhibiting its overlapping target enzymes, purified recombinant cystatin was used to test the inhibitory activity against papain-like cysteine proteases and caspase 1 as another family protease to verify HCcyst-3’s target specificity. The results showed that rHCcyst-3 effectively inhibited papain and cathepsin L, whereas the activity of cathepsin B was relatively less efficiently inhibited by rHCcyst-3, and rHCcyst-3 showed no inhibitory activity with caspase 1 (Fig. 4 and Table 1).

**Table 1:** Protease inhibition assays

Enzyme	Enzyme concentration (nM)	HCcyst-3 (nM)	
		IC_50_	95% CI
Papain	150	208.7	198.7–219.2
Cathepsin L	50	18.5	17.6–19.5
Cathepsin B	50	121.6	109.2–135.4
Caspase 1	50	NI	

*Abbreviation*: *CI*, confidence interval; *NI*, no inhibition

The concentration of HCcyst-3 at which 50% of the proteolytic enzymes’ activity is inhibited (IC_50_)

### Goat monocytes can uptake the rHCcyst-3

**Fig. 5:**
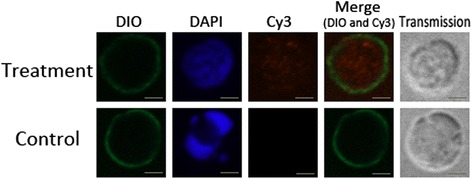
Uptake of rHCcyst-3 by goat monocytes. Goat monocytes were left untreated or incubated with rHCcyst-3 (40 μg/ml) for 30 min at 37 °C. All cells were fixed and incubated with rat anti-rHCcyst-3 antibody followed by Cy3 labeled goat anti-rat IgG (red). The nuclei and membranes of the corresponding cells were visualized by DAPI (blue) and DIO (green) staining, respectively. The internalization of rHCcyst-3 by goat monocytes was visualized with a confocal laser scanning microscopy. Merge, overlap of red and green channels. The data are representative of three independent experiments. *Scale-bars*: 2 μm

Goat monocytes were incubated with rHCcyst-3, and the protein uptake by monocytes was investigated by an immunofluorescence approach. As depicted in Fig. 5, the emission from the Cy3-labeled rHCcyst-3 was red, and the DAPI-labeled nuclei were blue and DIO-labeled cell membrane was green. No fluorescence was observed in any color channel in the unstained background control (not shown). In the control group, no red fluorescence was observed (Fig. 5 lower panel). Intense red fluorescence was observed when the cells were incubated with rHCcyst-3 (Fig. 5 upper panel). The results suggest that native HCcyst-3 protein can uptake by goat monocytes in vivo.

### The alteration of secreted cytokine levels

**Fig. 6:**
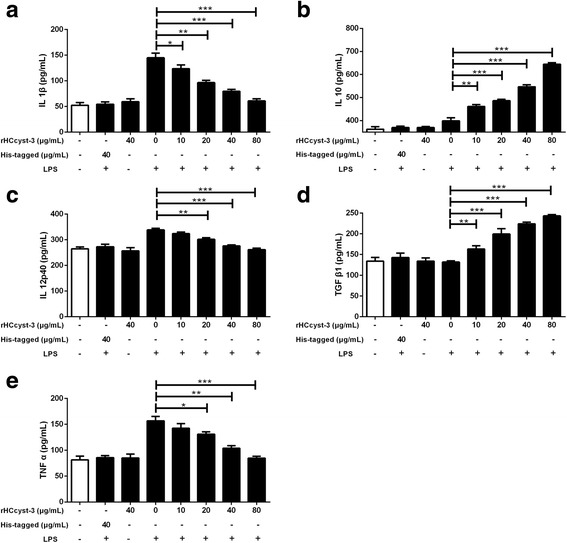
Regulation of cytokine secretion by rHCcyst-3. Goat monocytes were stimulated with LPS (100 ng/ml) for 72 h in the presence or absence of rHCcyst-3 and his-tagged protein. The cells treated with rHCcyst-3 alone as another control. Cytokine secretion in the supernatant of cell cultures was quantified by ELISA. **a** IL-1β. **b** IL-10. **c** IL-12p40. **d** TGF-β1. **e** TNF-α. The data are representative of three independent experiments (*P < 0.05, **P < 0.01, ***P < 0.001)

By performing ELISA, we noted that rHCcyst-3 decreased the LPS induced production of TNF-α (10 μg/ml: *t*_(4)_ = 2.003, *P* = 0.1158; 20 μg/ml: *t*_(4)_ = 4.602, *P* = 0.0100; 40 μg/ml: *t*_(4)_ = 7.580, *P* = 0.0016; 80 μg/ml: *t*_(4)_ = 11.65, *P* = 0.0003); IL-1β (10 μg/ml: *t*_(4)_ = 3.138, *P* = 0.0349; 20 μg/ml: *t*_(4)_ = 8.262, *P* = 0.0012; 40 μg/ml: *t*_(4)_ = 10.42, *P* = 0.0005; 80 μg/ml: *t*_(4)_ = 12.63, *P* = 0.0002); and IL-12p40 (10 μg/ml: *t*_(4)_ = 2.750, *P* = 0.0514; 20 μg/ml: *t*_(4)_ = 6.875, *P* = 0.0023; 40 μg/ml: *t*_(4)_ = 11.00, *P* = 0.0004; 80 μg/ml: *t*_(4)_ = 11.15, *P* = 0.0004) in goat monocytes. Intriguingly, rHCcyst-3 significantly increased the secretion of IL-10 (10 μg/ml: *t*_(4)_ = 7.225, *P* = 0.0019; 20 μg/ml: *t*_(4)_ = 10.38, *P* = 0.0005; 40 μg/ml: *t*_(4)_ = 12.63, *P* = 0.0002; 80 μg/ml: *t*_(4)_ = 25.39, *P* < 0.0001) and TGF-β1 (10 μg/ml: *t*_(4)_ = 6.718, *P* = 0.0026; 20 μg/ml: *t*_(4)_ = 8.720, *P* = 0.0010; 40 μg/ml: *t*_(4)_ = 21.90, *P* < 0.0001; 80 μg/ml: *t*_(4)_ = 29.99, *P* < 0.0001) in goat monocytes in a dose-dependent manner compared with LPS treated only (Fig. 6). The cells treated with his-tagged protein or rHCcyst-3 alone showed no significantly changed. The cytokines profile modulated by rHCcyst-3 contributes to induce an anti-inflammatory environment which favorable for the survival of worms.

### rHCcyst-3 inhibited MHC-II expression on goat monocytes

**Fig. 7:**
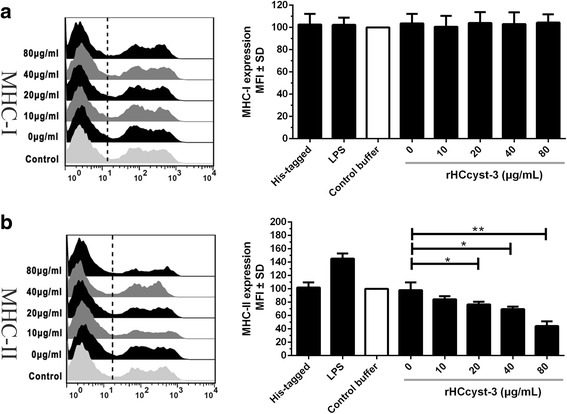
rHCcyst-3 inhibits MHC-II expression on goat monocytes. Monocytes were cultured in the presence of control buffer (PBS/DTT) or different concentrations of rHCcyst-3 and his-tagged protein for 24 h. The cells treated by LPS as the positive control. MHC-II expression was measured by flow cytometric analysis and calculated as the percentage of mean fluorescence intensity (MFI) of controls. Bars represent the MFI ± SD of controls. **a** MHC-I. **b** MHC-II. The data are representative of three independent experiments (*P < 0.05, **P < 0.01, ***P < 0.001)

Compared to the baseline expression of MHC-II in the control buffer, rHCcyst-3 significantly decreased MHC-II expression in a dose-dependent manner (10 μg/ml: *t*_(4)_ = 1.875, *P* = 0.1340; 20 μg/ml: *t*_(4)_ = 2.965, *P* = 0.0414; 40 μg/ml: *t*_(4)_ = 3.963, *P* = 0.0166; 80 μg/ml: *t*_(4)_ = 6.755, *P* = 0.0025) and no significant change in his-tagged protein treated group (Fig. 7). However, no changes were detected in MHC-I following exposure of goat monocytes to rHCcyst-3 at different concentrations (10 μg/ml: *t*_(4)_ = 0.4001, *P* = 0.7095; 20 μg/ml: *t*_(4)_ = 0.04411, *P* = 0.9669; 40 μg/ml: *t*_(4)_ = 0.08482, *P* = 0.9365; 80 μg/ml: *t*_(4)_ = 0.1010, *P* = 0.9244).

### NO production

**Fig. 8:**
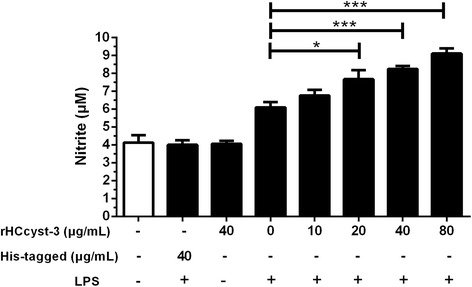
rHCcyst-3 enhance NO production on LPS treated goat monocytes. Monocytes were stimulated with LPS (100 ng/ml) for 48 h in the presence or absence of rHCcyst-3 and his-tagged protein. The cells treated with rHCcyst-3 alone as another control. NO was measured in the cell supernatants as nitrite using a NO assay kit. The data are representative of three independent experiments (*P < 0.05, **P < 0.01, ***P < 0.001)

The nitrate concentration of the culture supernatant was significantly increased by rHCcyst-3 in a dose-dependent manner (10 μg/ml: *t*_(4)_ = 2.697, *P* = 0.0543; 20 μg/ml: *t*_(4)_ = 4.565, *P* = 0.0103; 40 μg/ml: *t*_(4)_ = 8.630, *P* = 0.0010; 80 μg/ml: *t*_(4)_ = 8.911, *P* = 0.0009) and no significant change in his-tagged protein or rHCcyst-3 alone treated group was found. This suggested that rHCcyst-3 could up-regulate the LPS induced NO production of goat monocytes (Fig. 8).

### Capacity of phagocytosis

**Fig. 9:**
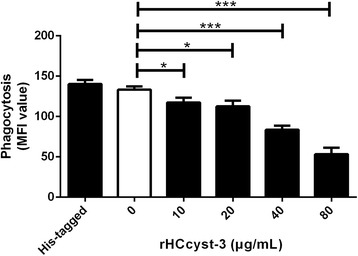
rHCcyst-3 decrease phagocytic capacity of goat monocytes. Monocytes were collected after treated with rHCcyst-3 or his-tagged protein for 48 h and incubated with FITC-dextran (1 mg/ml in RPMI1640) for 1 h at 37 °C. The FITC-dextran internalization of cells was analyzed by flow cytometry and calculated as mean fluorescence intensity (MFI). The data are representative of three independent experiments (*P < 0.05, **P < 0.01, ***P < 0.001)

Phagocytic capacity of goat monocytes after 48 h treatment with different concentrations of rHCcyst-3 was examined. As shown in Fig. 9, rHCcyst-3 significantly decreased the FITC-dextran uptake ability of goat monocytes in a dose-dependent manner (10 μg/ml: *t*_(4)_ = 3.819, *P* = 0.0188; 20 μg/ml: *t*_(4)_ = 4.384, *P* = 0.0118; 40 μg/ml: *t*_(4)_ = 13.33, *P* = 0.0002; 80 μg/ml: *t*_(4)_ = 15.43, *P* = 0.0001) and no significant change in his-tagged protein treated group. The results suggest that native HCcyst-3 protein can restrain phagocytic capacity of goat monocytes in vivo.

## Discussion

The capacity of helminth parasites to modulate the immune system underpins their longevity in the mammalian host [20]. There are several reports to show that nematode parasites that dwell in the gastrointestinal tract of their hosts can modulate the immune response systematically [21, 22]. Cystatins have been recognized as significant immune modulators associated with nematode parasites infection [11]. The target cells of cystatin-induced immunomodulation seem to be monocytes, as depletion of monocytes from the PBMC reversed the inhibitory effects of *O. volvulus* cystatin [23]. Here, we cloned a type 2 cystatin gene from *H. contortus*, HCcyst-3, produced recombinant HCcyst-3 protein and examined its immunomodulatory effects on goat monocytes.

The capacities of various cystatins to inhibit the activity of cysteine proteases have been characterized [24]. Here, the histidine-tagged fused recombinant cystatin efficiently inhibited the activity of cathepsin L, cathepsin B, and papain. rHCcyst-3 effectively inhibited cathepsin L, and it also had quite a high inhibitory efficiency against papain. However, rHCcyst-3 showed relatively less inhibitory activity against cathepsin B, perhaps due to an occluding loop in the cathepsin B active site that limits the access of both substrates and inhibitors to the active site [25]. The lack of inhibitory activity has been documented previously for other cystatins [21, 23, 24].

Different from type 1 cystatins, the type 2 cystatins are secretion-type proteins containing signal peptides. Type 2 cystatins from *Nippostrongylus brasiliensis*, *Heligmosomoides polygyrus*, *Litomosoides sigmodontis* and *Brugia malayi* were proved existence in excretory/secretory (ES) products of parasites [12, 21, 26, 27]. In the present study, we found that rHCcyst-3 could be recognized by the antiserum from goats experimentally infected with *H. contortus* and the native HCcyst-3 protein was predominantly localized at the body surface and internal surface of the parasite’s gut. Cystatins of helminth parasites play immune modulatory functions based on a hypothesis that host monocyte/macrophage can uptake parasite cystatins [28]. Furthermore, we demonstrated that goat monocyte could uptake rHCcyst-3 in vitro. These results indicated that type 2 cystatins of *H. contortus* were excretory/secretory antigens and interacted with the host immune system during infection. Theoretically, the immunomodulatory functions proposed to require constant secretion and certain concentrations of this molecule. However, HCcyst-3 accumulates to the functional concentration in vivo, so the real mechanism or pathways involved in host parasite interactions during natural infection of *H. contortus* are worthy for further studied.

Apart from their capacity to inhibit proteases, nematode cystatins seem to have a profound effect on the production of cytokines. In a series of experiments performed, filarial cystatins were shown to induce the production of several cytokines, which results in an anti-inflammatory response [11]. Schonemeyer et al. [23] demonstrated that *O. volvulus* cystatin induces an early TNF-α response in human PBMC, followed by a downregulation of the IL-12 production and a massive increase in IL-10 production of the cells. The IL-10 was mainly produced by monocytes as determined by purified human monocytes [23]. Antigen-specific production of IL-4 and IFN-γ from splenocytes was suppressed in mice treated with rNbCys compared to antigen-specific production in mice treated with control protein. The mice bone-marrow-derived dendritic cell generated in the presence of rHp-CPI exhibited reduced IL-6 and TNF-α cytokine production when stimulated with Toll-like receptor ligand CpG. Our results showed that rHCcyst-3 decreased the LPS induced production of TNF-α, IL-1β and IL-12p40 in goat monocytes. However, rHCcyst-3 significantly increased the secretion of IL-10 and TGF-β1 in goat monocytes in a dose-dependent manner compared with LPS treated only. The cytokines profile modulated by rHCcyst-3 contributes to induce an anti-inflammatory environment which favorable for the survival of worms.

NO is synthesized by NO synthase and has been identified as a major effector molecule released from activated macrophages [29, 30]. The diverse biological functions of NO were shown to reduce growth and inhibit invasiveness of protozoan parasites and regulate the innate immune response to the parasitic protozoa [31–34]. On the other hand, NO has been shown to induce a strong inhibition of lymphocyte proliferation in vitro and to regulate cytokine gene expression in various cell types [35, 36]. Here, rHCcyst-3 significantly enhanced the NO production by LPS treated goat monocytes. Previous studies have shown that other members of the cystatin superfamilies (i.e. chicken cystatin, human stefin B and rat T-kininogen) upregulate the NO production of IFN-γ-activated murine macrophages [30]. This feature is restricted to natural cysteine protease inhibitors, as synthetic inhibitors, like E64, fail to increase the NO production [37]. Interestingly, cystatins of nematodes, regardless of whether they are parasitic or free-living, share with other members of the cystatin superfamily potentially up-regulate the NO production of IFN-γ activated macrophages [6, 37, 38]. NO was associated with suppression of antigen-specificT cell proliferation in a murine model of filariasis [39]. Indeed, several studies revealed that parasitic nematode cystatins could inhibit antigen-specific T cell proliferation in vitro and in vivo [26, 38, 40]. The upregulation of NO production in LPS treated goat monocytes in the presence of rHCcyst-3 might suggest a T cell proliferation inhibition event occurs when HCcyst-3 release by parasites in parasitic process. However, we guess further study be needed.

Phagocytosis is an early and fundamental step for the effective clearance of disease causing agents. The phagocytotic function of phagocytes is an important indicator of the body’s immune competence [41]. The ability to engulf and kill pathogens is considered as a major effector function of macrophages [42]. In the present study, phagocytic capacity of goat monocytes was decreased after treatment with different concentrations of rHCcyst-3 in a dose-dependent manner.

Cysteine proteases in endosomes and lysosomes of antigen-presenting cells are known to be involved in the processing of protein antigens and MHC-II molecule maturation. Cathepsin S plays an important role in stepwise proteolytic degradation of the invariant chain (Ii) that regulates MHC-II molecule intracellular trafficking and protects the MHC-II molecule from premature binding of antigen peptide [43]. Cathepsin B and C are required for processing of antigen peptides and facilitate their binding to the MHC-II peptide binding groove [44]. It is reported that cystatin from *N. brasiliensis* inhibited the processing of OVA protein by lysosomal cysteine proteases from spleen cells of mice [21]. Furthermore, the presence of *O. volvulus* cystatin resulted in a reduction by 72% of human leucocyte antigen (HLA-DR) on human monocytes as compared to an irrelevant *O. volvulus* control protein. In the present study, we noted that rHCcyst-3 was able to inhibit MHC-II expression on goat monocytes in a dose dependent manner. However, no significant change of MHC-I expression was observed after exposure of rHCcyst-3.

## Conclusion

Our results showed that rHCcyst-3 could uptake by goat monocytes and exerts its immunomodulatory effects on multiple aspects to facilitate the immune evasion of *H. contortus.* These findings provide insight into the interactive relationship between parasitic nematode cystatins and host monocytes. It also sheds new light on the molecular mechanisms of helminthic immune evasion.

## Additional file

Additional file 1: Figure S1. The proteinase inhibition assays of his-tagged protein as control. (DOCX 162 kb)

## Abbreviations

DAB: Diaminobenzidine
DAPI: 2-(4-amidinophenyl)-6-indole carbamidinedihydrochloride
DIO: 3,3′-Dioctadecyloxacarbocyanine Perchlorate
DMEM: Dulbecco’s Modified Eagle Medium
ELISA: Enzyme-linked immunosorbent assay
EU: Endotoxin Unit
HCcyst-3: *Haemonchus contortus* cystatin 3
IL-10: Interleukin-10
IL-12p40: Interleukin-12p40
IL-1β: Interleukin-1β
IPTG: Isopropyl-β-D-thiogalactopyranoside
L3 s: Third-stage larvae
LAL: *Limulus* amoebocyte lysate
LPS: Lipophosphoglycan
MFI: Median fluorescence intensity
ORF: Open reading frame
PBMC: Peripheral blood mononuclear cells
PBS: Phosphate buffered saline
rHCcyst-3: Recombinant cystatins of *Haemonchus contortus* cystatin 3
RT-PCR: Reverse transcription polymerase chain reaction
SDS-PAGE: Sodium dodecyl sulfate polyacrylamide gel electrophoresis
TBS: Tris-buffered saline
TBST: Tris-buffered saline containing 0.1% Tween-20
TGF-β: Transforming growth factor-β
TNF-α: Tumor necrosis factor-α
