# Supplementary figures and images for: Characterization of a secreted cystatin of the parasitic nematode Haemonchus contortus and its immune-modulatory effect on goat monocytes
Source: Parasit Vectors. 2017 Sep 18;10:425. doi: 10.1186/s13071-017-2368-1 (PMC5604358; doi:10.1186/s13071-017-2368-1)

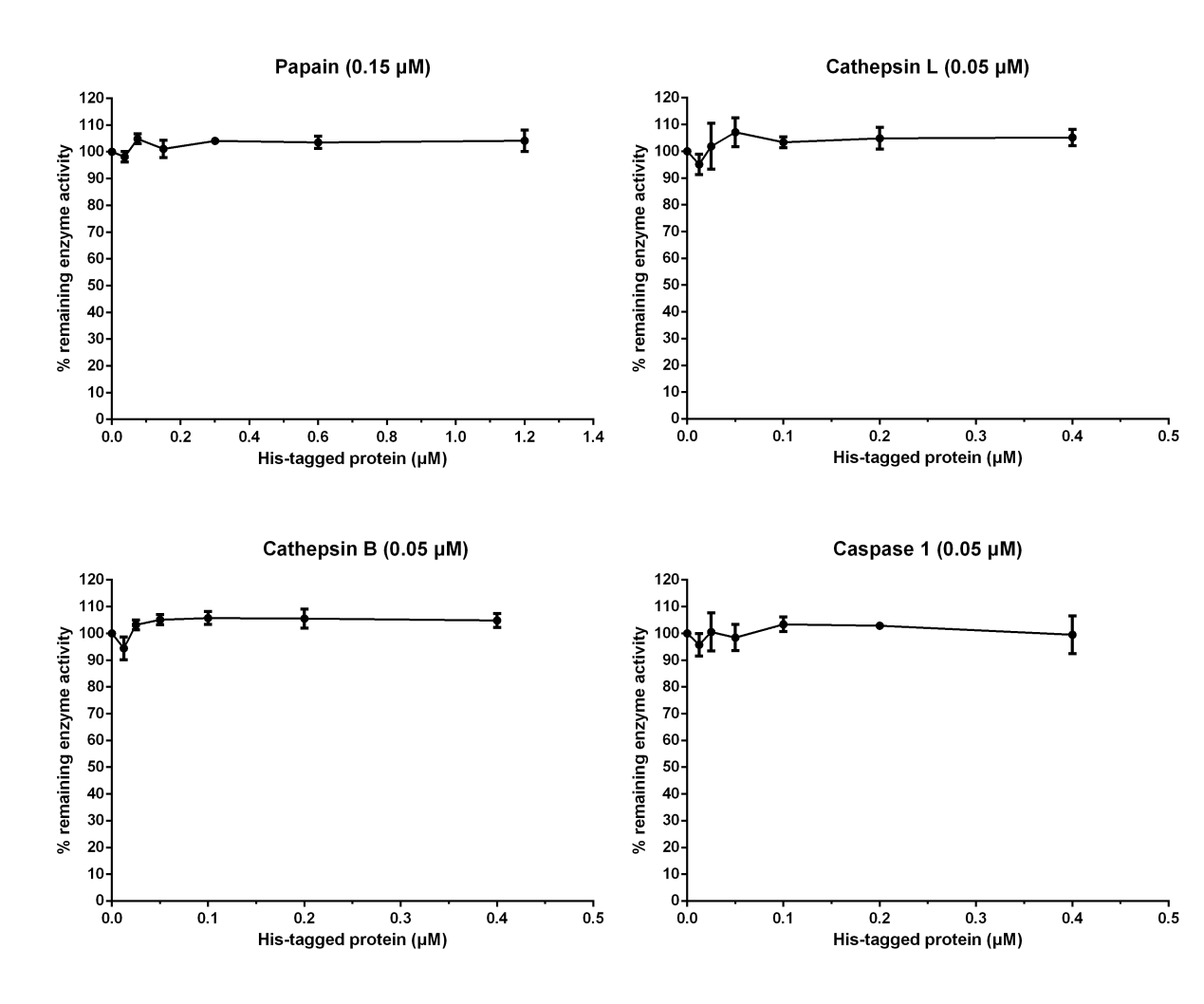


**Figure S1.** The proteinase inhibition assays of his-tagged protein as control.

Supplement: Additional file 1: Figure S1. — The proteinase inhibition assays of his-tagged protein as control. (DOCX 162 kb) [file 13071_2017_2368_MOESM1_ESM.docx]
